# Split hand/foot malformation type 1 associated with 7q21.3 deletion - a case report

**DOI:** 10.1186/1755-8166-7-S1-P57

**Published:** 2014-01-21

**Authors:** S Aswini, S Ambika, KS Pooja, D Anuradha, JS Kadandale, CR Samuel

**Affiliations:** 1 Department of Genetics, Dr. ALM PG. Institute of Basic Medical Sciences, University of Madras, Taramani, Chennai, Tamil Nadu, India; 2 Centre for Human Genetics, Bangalore, Karnataka, India; 3 Department of Medical Genetics, Institute of Obstetrics and Gynecology, Madras Medical College, Government Hospital for Women and Children, Egmore, Chennai , India

**Keywords:** SHFM1, Ectrodactyly, Developmental delay

## 

Split hand/split foot malformation (SHFM) is a rare congenital deformity involving limb development. SHFM, also known as ectrodactyly, is characterized by absence of digits, fusion of remaining digits, and a deep median cleft in the hands and feet. It has been observed to occur at a prevalence of approximately 1:18,000 newborns. This malformation is genetically heterogeneous involving several loci including 7q21-q22.1, Xq26, 10q24-q25, 2q31 and 3q27. New loci requiring further validation have also been suggested as valuable candidate regions.

Chromosomal rearrangements involving7q21-q22 is most commonly associated with isolated or syndromic ectrodactyly, referred to as SHFM type 1. We report a case of SHFM1 in an eight-month-old female baby with developmental delay. Follow up after two years revealed bilateral hearing loss also. Chromosome analysis using high resolution banding technique showed an interstitial deletion of the sub-band 7q21.3. FISH using BAC clones resolved the break to have occurred within the band 7q21.3. Cytogenetic evaluation of her parents showed the deletion to be of a de novo origin.

SFHM1 is expressed as an autosomal dominant trait with reduced penetrance and variable expression and is accompanied by deafness in 35% of the patients as observed in the proposita. Several studies have pointed out the probable role of three genes present in this region – DLX5, DLX6 and DSS1 - in limb development.

This report reiterates the importance of high resolution banding and molecular cytogenetic techniques such as FISH in the detection and delineation of subtle abnormalities. Array CGH will help in further refining the deleted region and thus in the discovery of candidate genes for the phenotypic characteristics.

